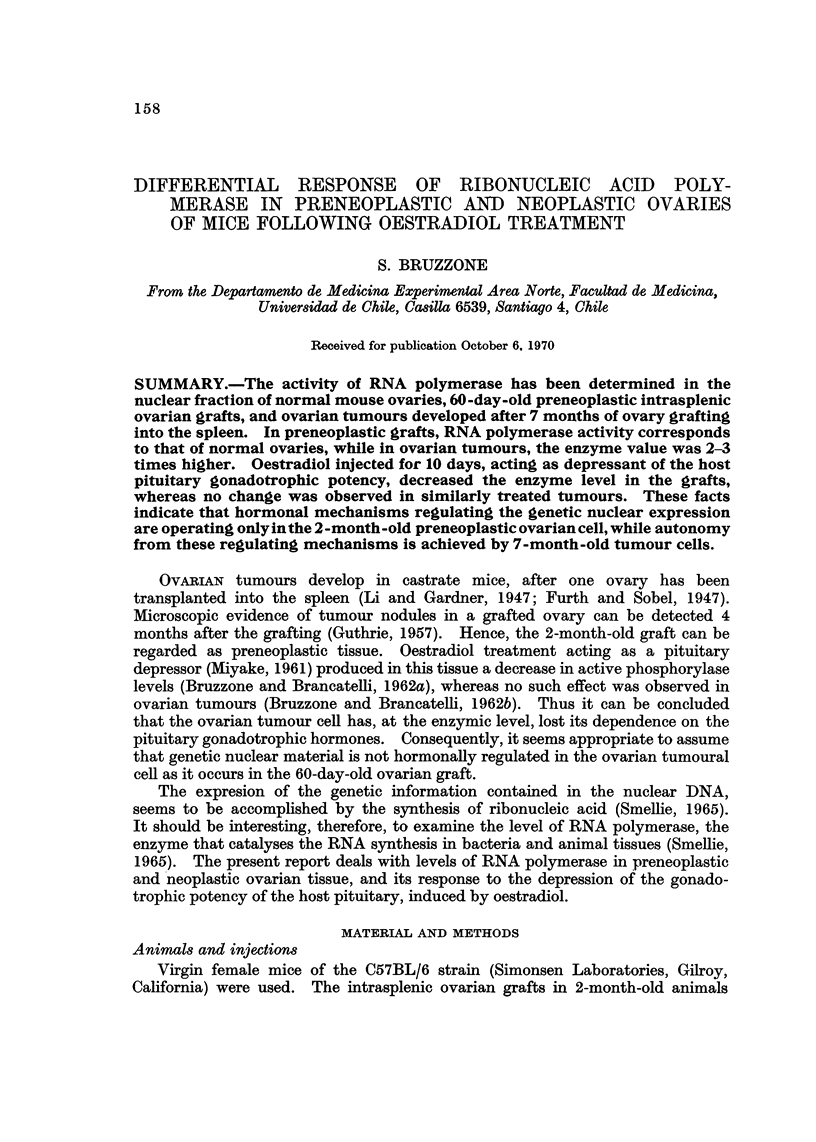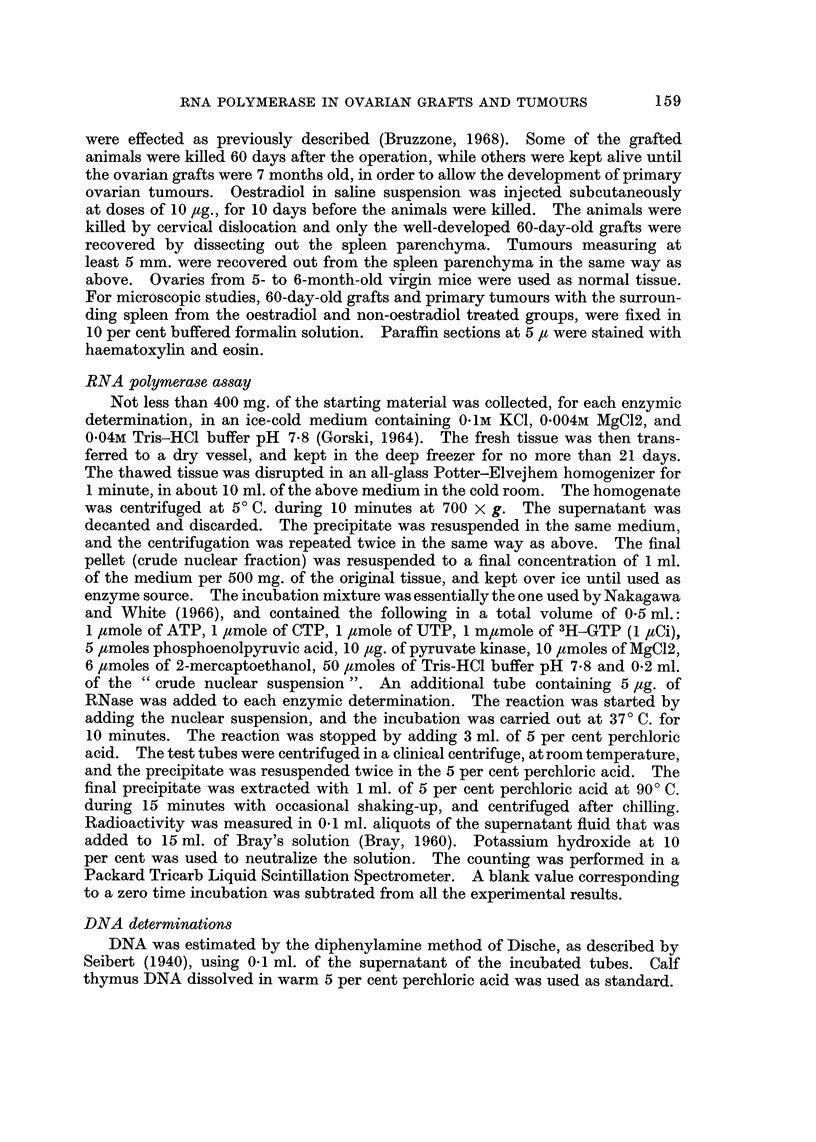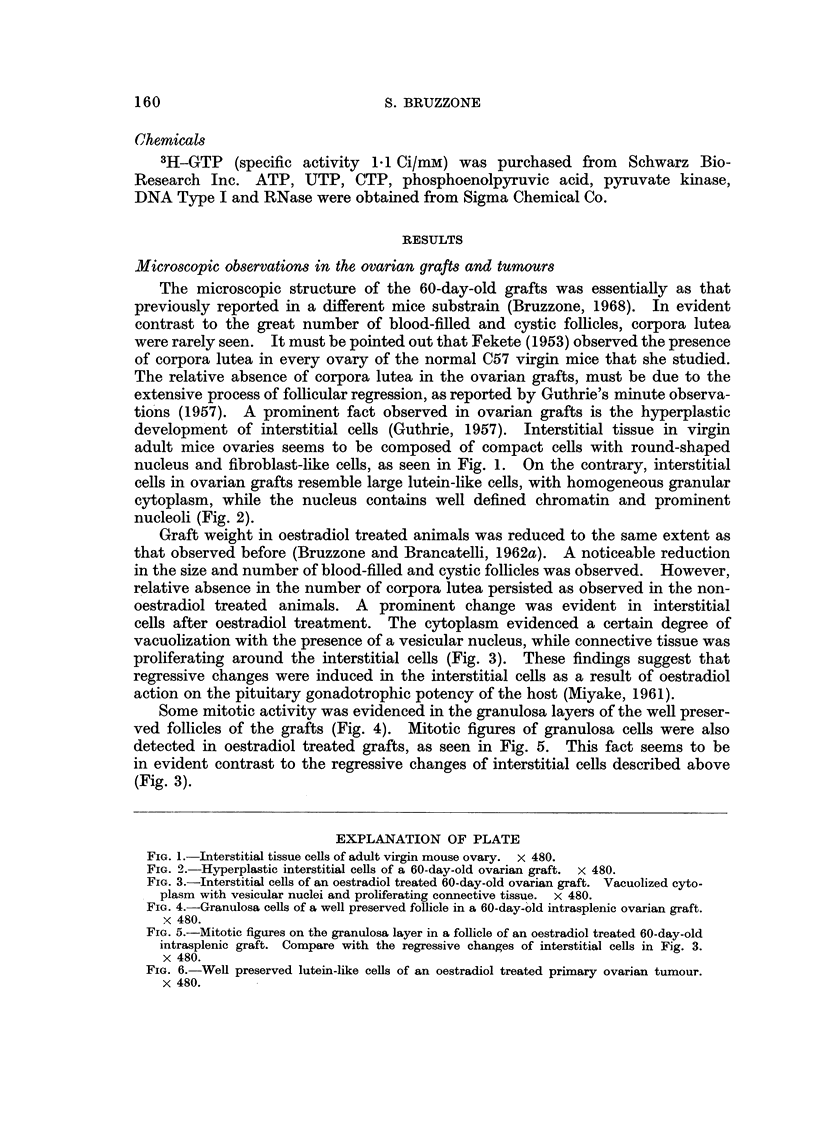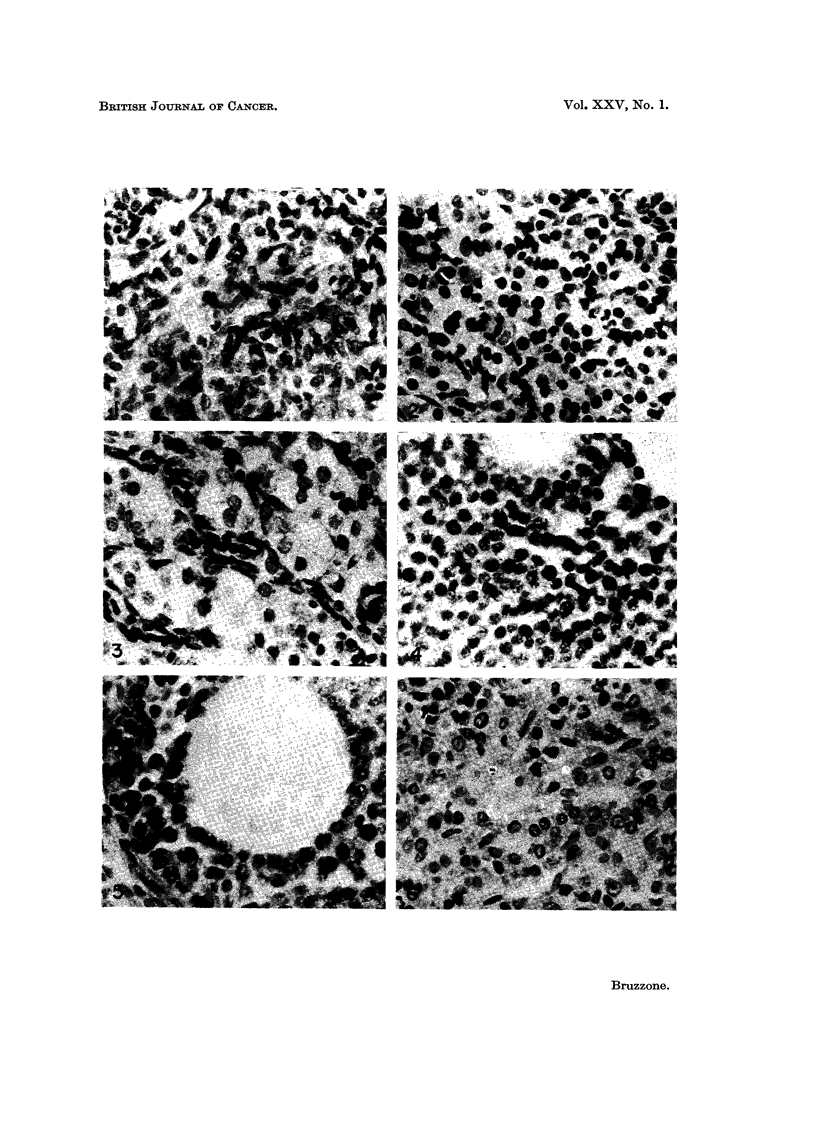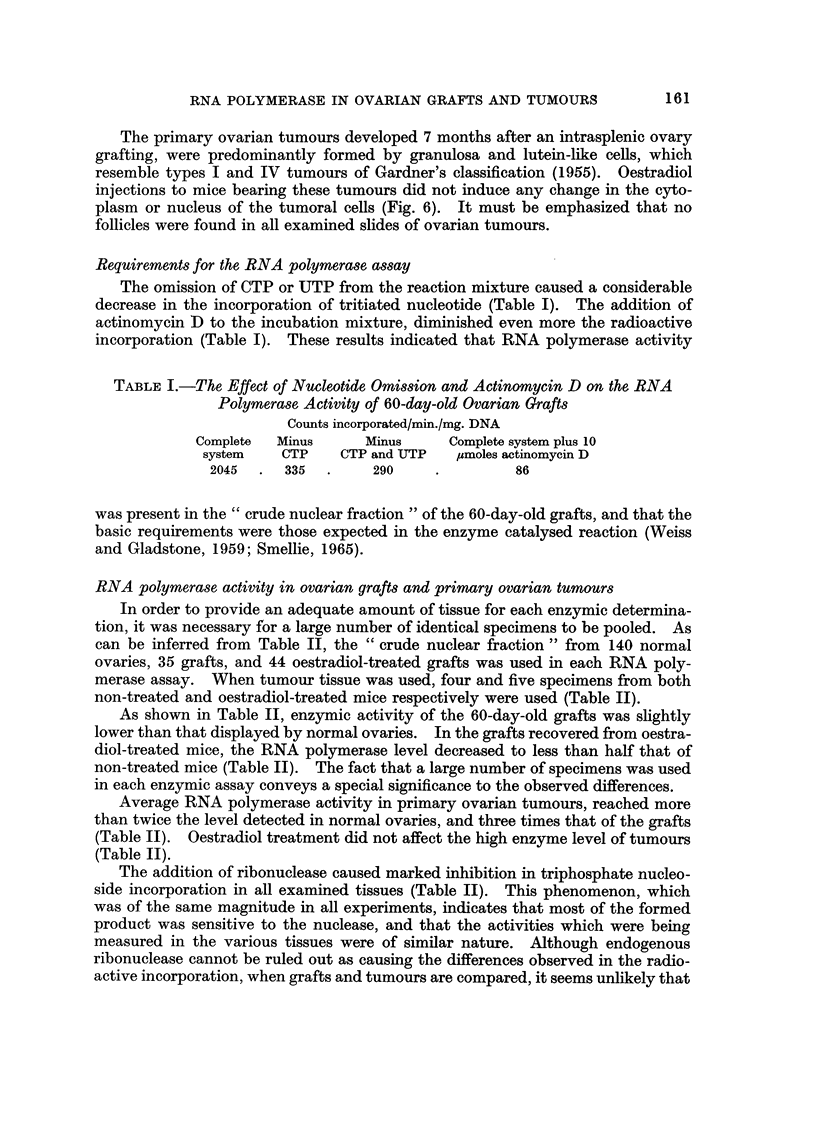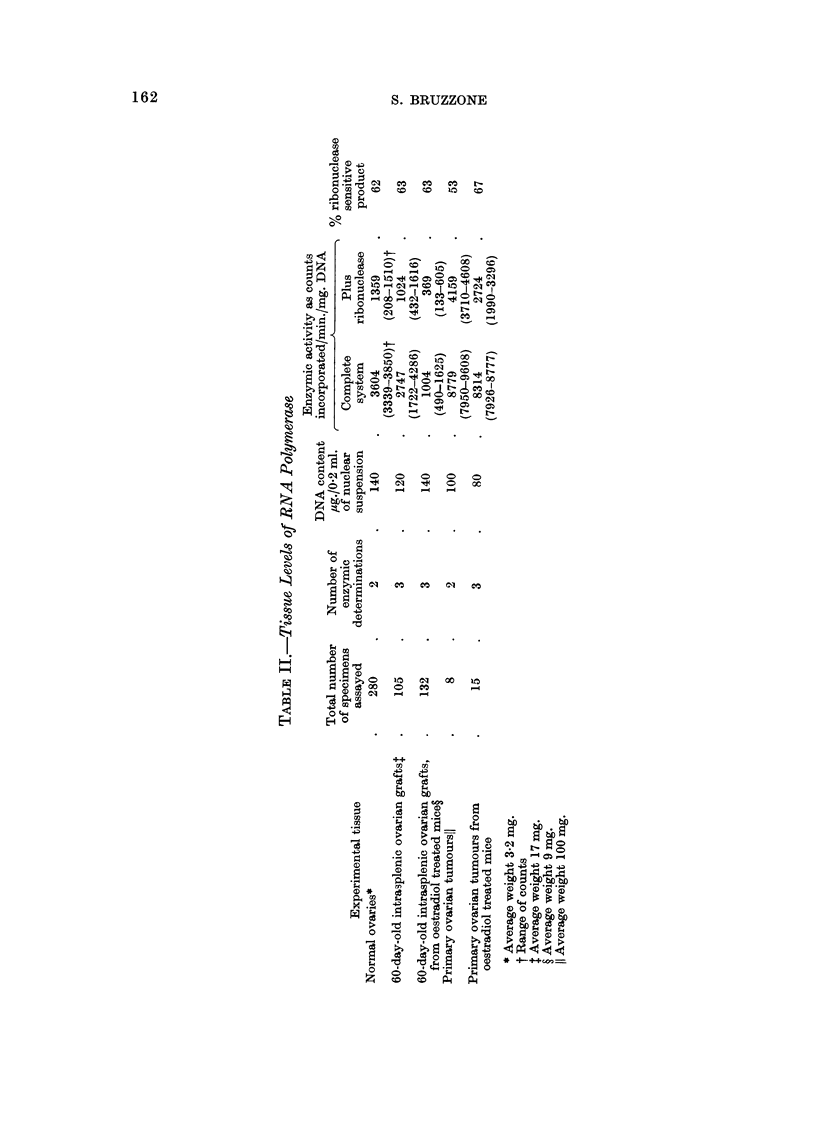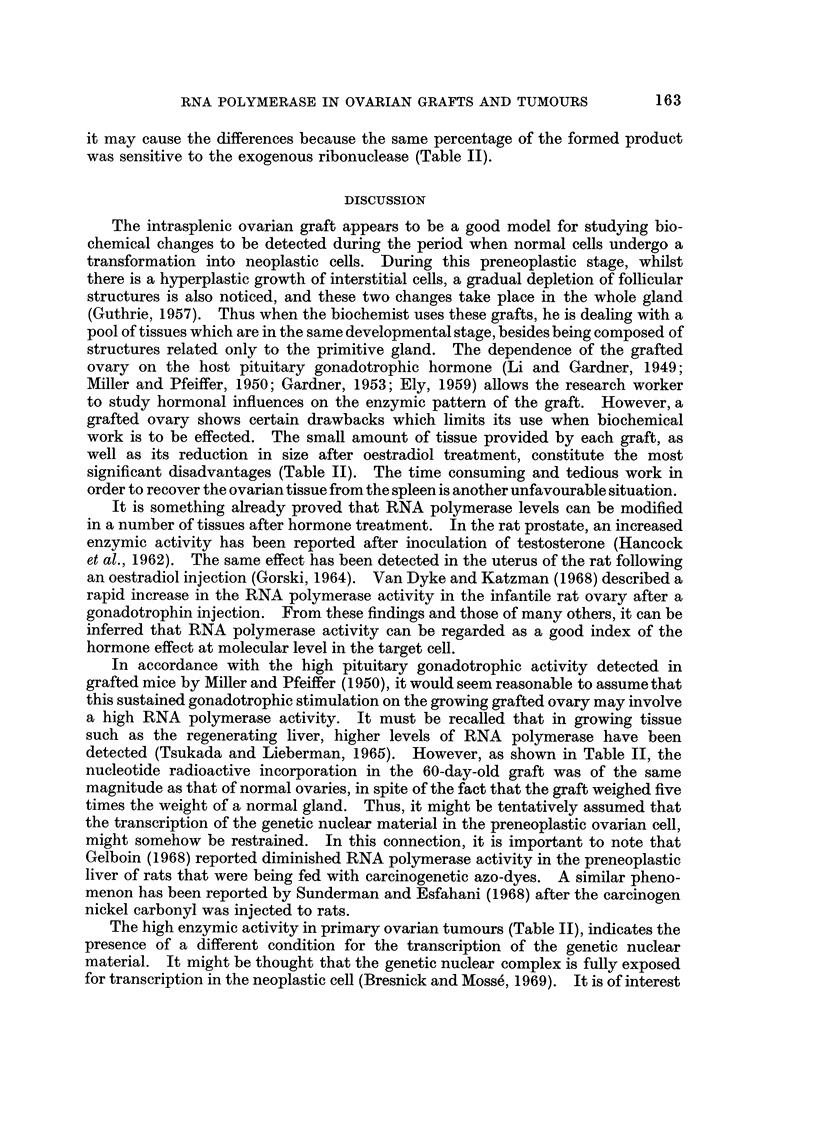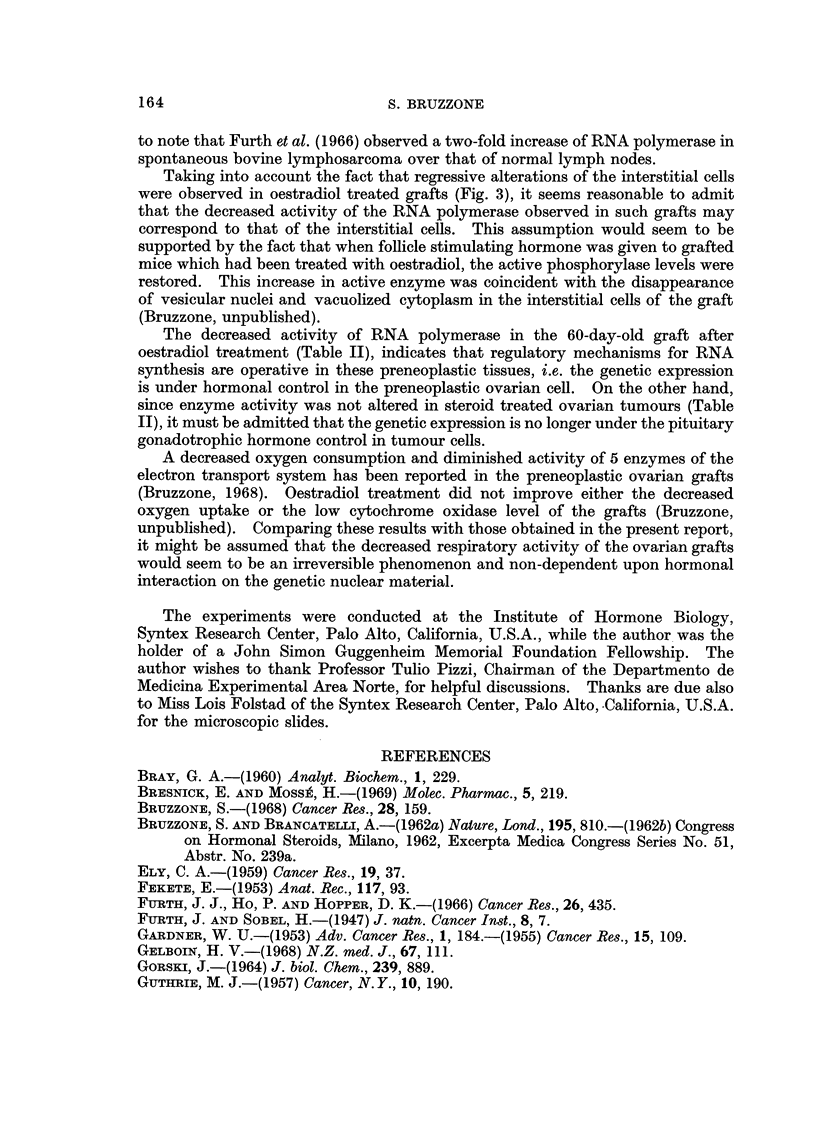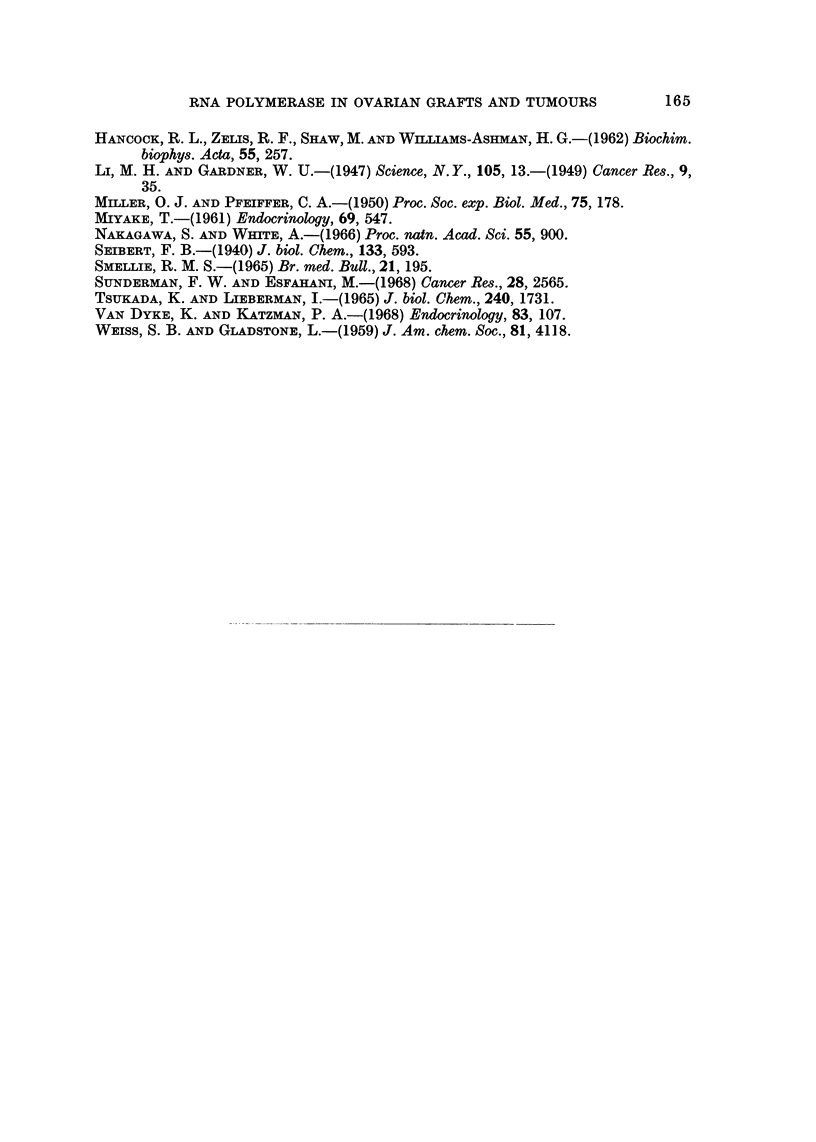# Differential Response of Ribonucleic Acid Polymerase in Preneoplastic and Neoplastic Ovaries of Mice Following Oestradiol Treatment

**DOI:** 10.1038/bjc.1971.22

**Published:** 1971-03

**Authors:** S. Bruzzone

## Abstract

**Images:**


### DIFFERENTIAL RESPONSE OF RIBONUCLEIC ACID POLYMERASE IN PRENEOPLASTIC AND NEOPLASTIC OVARIES OF MICE FOLLOWING OESTRADIOL TREATMENT

S. BRUZZONE

*From the Departamento de Medicina Experimental Area Norte, Facultad de Medicina, Universidad de Chile, Casilla 6539, Santiago 4, Chile*

Received for publication October 6, 1970

**SUMMARY.—The activity of RNA polymerase has been determined in the nuclear fraction of normal mouse ovaries, 60-day-old preneoplastic intrasplenic ovarian grafts, and ovarian tumours developed after 7 months of ovary grafting into the spleen. In preneoplastic grafts, RNA polymerase activity corresponds to that of normal ovaries, while in ovarian tumours, the enzyme value was 2–3 times higher. Oestradiol injected for 10 days, acting as depressant of the host pituitary gonadotrophic potency, decreased the enzyme level in the grafts, whereas no change was observed in similarly treated tumours. These facts indicate that hormonal mechanisms regulating the genetic nuclear expression are operating only in the 2-month-old preneoplastic ovarian cell, while autonomy from these regulating mechanisms is achieved by 7-month-old tumour cells.**

OVARIAN tumours develop in castrate mice, after one ovary has been transplanted into the spleen (Li and Gardner, 1947; Furth and Sobel, 1947). Microscopic evidence of tumour nodules in a grafted ovary can be detected 4 months after the grafting (Guthrie, 1957). Hence, the 2-month-old graft can be regarded as preneoplastic tissue. Oestradiol treatment acting as a pituitary depressor (Miyake, 1961) produced in this tissue a decrease in active phosphorylase levels (Bruzzone and Brancatelli, 1962*a*), whereas no such effect was observed in ovarian tumours (Bruzzone and Brancatelli, 1962*b*). Thus it can be concluded that the ovarian tumour cell has, at the enzymic level, lost its dependence on the pituitary gonadotrophic hormones. Consequently, it seems appropriate to assume that genetic nuclear material is not hormonally regulated in the ovarian tumoural cell as it occurs in the 60-day-old ovarian graft.

The expresion of the genetic information contained in the nuclear DNA, seems to be accomplished by the synthesis of ribonucleic acid (Smellie, 1965). It should be interesting, therefore, to examine the level of RNA polymerase, the enzyme that catalyses the RNA synthesis in bacteria and animal tissues (Smellie, 1965). The present report deals with levels of RNA polymerase in preneoplastic and neoplastic ovarian tissue, and its response to the depression of the gonadotrophic potency of the host pituitary, induced by oestradiol.

### MATERIAL AND METHODS

### *Animals and injections*

Virgin female mice of the C57BL/6 strain (Simonsen Laboratories, Gilroy, California) were used. The intrasplenic ovarian grafts in 2-month-old animals were effected as previously described (Bruzzone, 1968). Some of the grafted animals were killed 60 days after the operation, while others were kept alive until the ovarian grafts were 7 months old, in order to allow the development of primary ovarian tumours. Oestradiol in saline suspension was injected subcutaneously at doses of 10 $\mu$g., for 10 days before the animals were killed. The animals were killed by cervical dislocation and only the well-developed 60-day-old grafts were recovered by dissecting out the spleen parenchyma. Tumours measuring at least 5 mm. were recovered out from the spleen parenchyma in the same way as above. Ovaries from 5- to 6-month-old virgin mice were used as normal tissue. For microscopic studies, 60-day-old grafts and primary tumours with the surrounding spleen from the oestradiol and non-oestradiol treated groups, were fixed in 10 per cent buffered formalin solution. Paraffin sections at 5 $\mu$ were stained with haematoxylin and eosin.

*RNA polymerase assay*

Not less than 400 mg. of the starting material was collected, for each enzymic determination, in an ice-cold medium containing 0·1M KCl, 0·004M MgCl2, and 0·04M Tris–HCl buffer pH 7·8 (Gorski, 1964). The fresh tissue was then transferred to a dry vessel, and kept in the deep freezer for no more than 21 days. The thawed tissue was disrupted in an all-glass Potter–Elvejhem homogenizer for 1 minute, in about 10 ml. of the above medium in the cold room. The homogenate was centrifuged at 5° C. during 10 minutes at 700 $\times$ ***g***. The supernatant was decanted and discarded. The precipitate was resuspended in the same medium, and the centrifugation was repeated twice in the same way as above. The final pellet (crude nuclear fraction) was resuspended to a final concentration of 1 ml. of the medium per 500 mg. of the original tissue, and kept over ice until used as enzyme source. The incubation mixture was essentially the one used by Nakagawa and White (1966), and contained the following in a total volume of 0·5 ml.: 1 $\mu$mole of ATP, 1 $\mu$mole of CTP, 1 $\mu$mole of UTP, 1 m$\mu$mole of $^3$H–GTP (1 $\mu$Ci), 5 $\mu$moles phosphoenolpyruvic acid, 10 $\mu$g. of pyruvate kinase, 10 $\mu$moles of MgCl2, 6 $\mu$moles of 2-mercaptoethanol, 50 $\mu$moles of Tris-HCl buffer pH 7·8 and 0·2 ml. of the " crude nuclear suspension ". An additional tube containing 5 $\mu$g. of RNase was added to each enzymic determination. The reaction was started by adding the nuclear suspension, and the incubation was carried out at 37° C. for 10 minutes. The reaction was stopped by adding 3 ml. of 5 per cent perchloric acid. The test tubes were centrifuged in a clinical centrifuge, at room temperature, and the precipitate was resuspended twice in the 5 per cent perchloric acid. The final precipitate was extracted with 1 ml. of 5 per cent perchloric acid at 90° C. during 15 minutes with occasional shaking-up, and centrifuged after chilling. Radioactivity was measured in 0·1 ml. aliquots of the supernatant fluid that was added to 15 ml. of Bray's solution (Bray, 1960). Potassium hydroxide at 10 per cent was used to neutralize the solution. The counting was performed in a Packard Tricarb Liquid Scintillation Spectrometer. A blank value corresponding to a zero time incubation was subtrated from all the experimental results.

*DNA determinations*

DNA was estimated by the diphenylamine method of Dische, as described by Seibert (1940), using 0·1 ml. of the supernatant of the incubated tubes. Calf thymus DNA dissolved in warm 5 per cent perchloric acid was used as standard.

*Chemicals*

$^3$H–GTP (specific activity 1·1 Ci/mM) was purchased from Schwarz Bio-Research Inc. ATP, UTP, CTP, phosphoenolpyruvic acid, pyruvate kinase, DNA Type I and RNase were obtained from Sigma Chemical Co.

### RESULTS

*Microscopic observations in the ovarian grafts and tumours*

The microscopic structure of the 60-day-old grafts was essentially as that previously reported in a different mice substrain (Bruzzone, 1968). In evident contrast to the great number of blood-filled and cystic follicles, corpora lutea were rarely seen. It must be pointed out that Fekete (1953) observed the presence of corpora lutea in every ovary of the normal C57 virgin mice that she studied. The relative absence of corpora lutea in the ovarian grafts, must be due to the extensive process of follicular regression, as reported by Guthrie's minute observations (1957). A prominent fact observed in ovarian grafts is the hyperplastic development of interstitial cells (Guthrie, 1957). Interstitial tissue in virgin adult mice ovaries seems to be composed of compact cells with round-shaped nucleus and fibroblast-like cells, as seen in Fig. 1. On the contrary, interstitial cells in ovarian grafts resemble large lutein-like cells, with homogeneous granular cytoplasm, while the nucleus contains well defined chromatin and prominent nucleoli (Fig. 2).

Graft weight in oestradiol treated animals was reduced to the same extent as that observed before (Bruzzone and Brancatelli, 1962*a*). A noticeable reduction in the size and number of blood-filled and cystic follicles was observed. However, relative absence in the number of corpora lutea persisted as observed in the non-oestradiol treated animals. A prominent change was evident in interstitial cells after oestradiol treatment. The cytoplasm evidenced a certain degree of vacuolization with the presence of a vesicular nucleus, while connective tissue was proliferating around the interstitial cells (Fig. 3). These findings suggest that regressive changes were induced in the interstitial cells as a result of oestradiol action on the pituitary gonadotrophic potency of the host (Miyake, 1961).

Some mitotic activity was evidenced in the granulosa layers of the well preserved follicles of the grafts (Fig. 4). Mitotic figures of granulosa cells were also detected in oestradiol treated grafts, as seen in Fig. 5. This fact seems to be in evident contrast to the regressive changes of interstitial cells described above (Fig. 3).

---

### EXPLANATION OF PLATE

FIG. 1.—Interstitial tissue cells of adult virgin mouse ovary. × 480.

FIG. 2.—Hyperplastic interstitial cells of a 60-day-old ovarian graft. × 480.

FIG. 3.—Interstitial cells of an oestradiol treated 60-day-old ovarian graft. Vacuolized cytoplasm with vesicular nuclei and proliferating connective tissue. × 480.

FIG. 4.—Granulosa cells of a well preserved follicle in a 60-day-old intrasplenic ovarian graft. × 480.

FIG. 5.—Mitotic figures on the granulosa layer in a follicle of an oestradiol treated 60-day-old intrasplenic graft. Compare with the regressive changes of interstitial cells in Fig. 3. × 480.

FIG. 6.—Well preserved lutein-like cells of an oestradiol treated primary ovarian tumour. × 480.

Bruzzone.

The primary ovarian tumours developed 7 months after an intrasplenic ovary grafting, were predominantly formed by granulosa and lutein-like cells, which resemble types I and IV tumours of Gardner's classification (1955). Oestradiol injections to mice bearing these tumours did not induce any change in the cytoplasm or nucleus of the tumoral cells (Fig. 6). It must be emphasized that no follicles were found in all examined slides of ovarian tumours.

*Requirements for the RNA polymerase assay*

The omission of CTP or UTP from the reaction mixture caused a considerable decrease in the incorporation of tritiated nucleotide (Table I). The addition of actinomycin D to the incubation mixture, diminished even more the radioactive incorporation (Table I). These results indicated that RNA polymerase activity

TABLE I.—*The Effect of Nucleotide Omission and Actinomycin D on the RNA Polymerase Activity of 60-day-old Ovarian Grafts*

Counts incorporated/min./mg. DNA

| Complete system | Minus CTP | Minus CTP and UTP | Complete system plus 10 μmoles actinomycin D |
|---|---|---|---|
| 2045 | 335 | 290 | 86 |

was present in the " crude nuclear fraction " of the 60-day-old grafts, and that the basic requirements were those expected in the enzyme catalysed reaction (Weiss and Gladstone, 1959; Smellie, 1965).

*RNA polymerase activity in ovarian grafts and primary ovarian tumours*

In order to provide an adequate amount of tissue for each enzymic determination, it was necessary for a large number of identical specimens to be pooled. As can be inferred from Table II, the " crude nuclear fraction " from 140 normal ovaries, 35 grafts, and 44 oestradiol-treated grafts was used in each RNA polymerase assay. When tumour tissue was used, four and five specimens from both non-treated and oestradiol-treated mice respectively were used (Table II).

As shown in Table II, enzymic activity of the 60-day-old grafts was slightly lower than that displayed by normal ovaries. In the grafts recovered from oestradiol-treated mice, the RNA polymerase level decreased to less than half that of non-treated mice (Table II). The fact that a large number of specimens was used in each enzymic assay conveys a special significance to the observed differences.

Average RNA polymerase activity in primary ovarian tumours, reached more than twice the level detected in normal ovaries, and three times that of the grafts (Table II). Oestradiol treatment did not affect the high enzyme level of tumours (Table II).

The addition of ribonuclease caused marked inhibition in triphosphate nucleoside incorporation in all examined tissues (Table II). This phenomenon, which was of the same magnitude in all experiments, indicates that most of the formed product was sensitive to the nuclease, and that the activities which were being measured in the various tissues were of similar nature. Although endogenous ribonuclease cannot be ruled out as causing the differences observed in the radioactive incorporation, when grafts and tumours are compared, it seems unlikely that

TABLE II.—*Tissue Levels of RNA Polymerase*

| Experimental tissue | Total number of specimens assayed | Number of enzymic determinations | DNA content $\mu$g./0·2 ml. of nuclear suspension | Enzymic activity as counts incorporated/min./mg. DNA: Complete system | Enzymic activity as counts incorporated/min./mg. DNA: Plus ribonuclease | % ribonuclease sensitive product |
|---|---|---|---|---|---|---|
| Normal ovaries* | 280 | 2 | 140 | 3604 (3339–3850)† | 1359 (208–1510)† | 62 |
| 60-day-old intrasplenic ovarian grafts‡ | 105 | 3 | 120 | 2747 (1722–4286) | 1024 (432–1616) | 63 |
| 60-day-old intrasplenic ovarian grafts, from oestradiol treated mice§ | 132 | 3 | 140 | 1004 (490–1625) | 369 (133–605) | 63 |
| Primary ovarian tumours‖ | 8 | 2 | 100 | 8779 (7950–9608) | 4159 (3710–4608) | 53 |
| Primary ovarian tumours from oestradiol treated mice | 15 | 3 | 80 | 8314 (7926–8777) | 2724 (1990–3296) | 67 |

* Average weight 3·2 mg.
† Range of counts
‡ Average weight 17 mg.
§ Average weight 9 mg.
‖ Average weight 100 mg.

it may cause the differences because the same percentage of the formed product was sensitive to the exogenous ribonuclease (Table II).

### DISCUSSION

The intrasplenic ovarian graft appears to be a good model for studying biochemical changes to be detected during the period when normal cells undergo a transformation into neoplastic cells. During this preneoplastic stage, whilst there is a hyperplastic growth of interstitial cells, a gradual depletion of follicular structures is also noticed, and these two changes take place in the whole gland (Guthrie, 1957). Thus when the biochemist uses these grafts, he is dealing with a pool of tissues which are in the same developmental stage, besides being composed of structures related only to the primitive gland. The dependence of the grafted ovary on the host pituitary gonadotrophic hormone (Li and Gardner, 1949; Miller and Pfeiffer, 1950; Gardner, 1953; Ely, 1959) allows the research worker to study hormonal influences on the enzymic pattern of the graft. However, a grafted ovary shows certain drawbacks which limits its use when biochemical work is to be effected. The small amount of tissue provided by each graft, as well as its reduction in size after oestradiol treatment, constitute the most significant disadvantages (Table II). The time consuming and tedious work in order to recover the ovarian tissue from the spleen is another unfavourable situation.

It is something already proved that RNA polymerase levels can be modified in a number of tissues after hormone treatment. In the rat prostate, an increased enzymic activity has been reported after inoculation of testosterone (Hancock *et al.*, 1962). The same effect has been detected in the uterus of the rat following an oestradiol injection (Gorski, 1964). Van Dyke and Katzman (1968) described a rapid increase in the RNA polymerase activity in the infantile rat ovary after a gonadotrophin injection. From these findings and those of many others, it can be inferred that RNA polymerase activity can be regarded as a good index of the hormone effect at molecular level in the target cell.

In accordance with the high pituitary gonadotrophic activity detected in grafted mice by Miller and Pfeiffer (1950), it would seem reasonable to assume that this sustained gonadotrophic stimulation on the growing grafted ovary may involve a high RNA polymerase activity. It must be recalled that in growing tissue such as the regenerating liver, higher levels of RNA polymerase have been detected (Tsukada and Lieberman, 1965). However, as shown in Table II, the nucleotide radioactive incorporation in the 60-day-old graft was of the same magnitude as that of normal ovaries, in spite of the fact that the graft weighed five times the weight of a normal gland. Thus, it might be tentatively assumed that the transcription of the genetic nuclear material in the preneoplastic ovarian cell, might somehow be restrained. In this connection, it is important to note that Gelboin (1968) reported diminished RNA polymerase activity in the preneoplastic liver of rats that were being fed with carcinogenetic azo-dyes. A similar phenomenon has been reported by Sunderman and Esfahani (1968) after the carcinogen nickel carbonyl was injected to rats.

The high enzymic activity in primary ovarian tumours (Table II), indicates the presence of a different condition for the transcription of the genetic nuclear material. It might be thought that the genetic nuclear complex is fully exposed for transcription in the neoplastic cell (Bresnick and Mossé, 1969). It is of interest to note that Furth *et al.* (1966) observed a two-fold increase of RNA polymerase in spontaneous bovine lymphosarcoma over that of normal lymph nodes.

Taking into account the fact that regressive alterations of the interstitial cells were observed in oestradiol treated grafts (Fig. 3), it seems reasonable to admit that the decreased activity of the RNA polymerase observed in such grafts may correspond to that of the interstitial cells. This assumption would seem to be supported by the fact that when follicle stimulating hormone was given to grafted mice which had been treated with oestradiol, the active phosphorylase levels were restored. This increase in active enzyme was coincident with the disappearance of vesicular nuclei and vacuolized cytoplasm in the interstitial cells of the graft (Bruzzone, unpublished).

The decreased activity of RNA polymerase in the 60-day-old graft after oestradiol treatment (Table II), indicates that regulatory mechanisms for RNA synthesis are operative in these preneoplastic tissues, *i.e.* the genetic expression is under hormonal control in the preneoplastic ovarian cell. On the other hand, since enzyme activity was not altered in steroid treated ovarian tumours (Table II), it must be admitted that the genetic expression is no longer under the pituitary gonadotrophic hormone control in tumour cells.

A decreased oxygen consumption and diminished activity of 5 enzymes of the electron transport system has been reported in the preneoplastic ovarian grafts (Bruzzone, 1968). Oestradiol treatment did not improve either the decreased oxygen uptake or the low cytochrome oxidase level of the grafts (Bruzzone, unpublished). Comparing these results with those obtained in the present report, it might be assumed that the decreased respiratory activity of the ovarian grafts would seem to be an irreversible phenomenon and non-dependent upon hormonal interaction on the genetic nuclear material.

The experiments were conducted at the Institute of Hormone Biology, Syntex Research Center, Palo Alto, California, U.S.A., while the author was the holder of a John Simon Guggenheim Memorial Foundation Fellowship. The author wishes to thank Professor Tulio Pizzi, Chairman of the Departmento de Medicina Experimental Area Norte, for helpful discussions. Thanks are due also to Miss Lois Folstad of the Syntex Research Center, Palo Alto, California, U.S.A. for the microscopic slides.

### REFERENCES

Bray, G. A.—(1960) *Analyt. Biochem.*, **1**, 229.
Bresnick, E. and Mossé, H.—(1969) *Molec. Pharmac.*, **5**, 219.
Bruzzone, S.—(1968) *Cancer Res.*, **28**, 159.
Bruzzone, S. and Brancatelli, A.—(1962*a*) *Nature, Lond.*, **195**, 810.—(1962*b*) Congress on Hormonal Steroids, Milano, 1962, Excerpta Medica Congress Series No. 51, Abstr. No. 239a.
Ely, C. A.—(1959) *Cancer Res.*, **19**, 37.
Fekete, E.—(1953) *Anat. Rec.*, **117**, 93.
Furth, J. J., Ho, P. and Hopper, D. K.—(1966) *Cancer Res.*, **26**, 435.
Furth, J. and Sobel, H.—(1947) *J. natn. Cancer Inst.*, **8**, 7.
Gardner, W. U.—(1953) *Adv. Cancer Res.*, **1**, 184.—(1955) *Cancer Res.*, **15**, 109.
Gelboin, H. V.—(1968) *N.Z. med. J.*, **67**, 111.
Gorski, J.—(1964) *J. biol. Chem.*, **239**, 889.
Guthrie, M. J.—(1957) *Cancer, N.Y.*, **10**, 190.

HANCOCK, R. L., ZELIS, R. F., SHAW, M. AND WILLIAMS-ASHMAN, H. G.—(1962) *Biochim. biophys. Acta*, **55**, 257.
LI, M. H. AND GARDNER, W. U.—(1947) *Science, N.Y.*, **105**, 13.—(1949) *Cancer Res.*, **9**, 35.
MILLER, O. J. AND PFEIFFER, C. A.—(1950) *Proc. Soc. exp. Biol. Med.*, **75**, 178.
MIYAKE, T.—(1961) *Endocrinology*, **69**, 547.
NAKAGAWA, S. AND WHITE, A.—(1966) *Proc. natn. Acad. Sci.* **55**, 900.
SEIBERT, F. B.—(1940) *J. biol. Chem.*, **133**, 593.
SMELLIE, R. M. S.—(1965) *Br. med. Bull.*, **21**, 195.
SUNDERMAN, F. W. AND ESFAHANI, M.—(1968) *Cancer Res.*, **28**, 2565.
TSUKADA, K. AND LIEBERMAN, I.—(1965) *J. biol. Chem.*, **240**, 1731.
VAN DYKE, K. AND KATZMAN, P. A.—(1968) *Endocrinology*, **83**, 107.
WEISS, S. B. AND GLADSTONE, L.—(1959) *J. Am. chem. Soc.*, **81**, 4118.